# Progress in State Medicine

**Published:** 1903-05-30

**Authors:** 


### Progress in State Medicine.

*(Concluded from page 137.)*

### SMALL-POX.

McVail,[4] seeing that the Vaccination Act of 1898 comes to an end in December, 1903, thinks that the passing of a Revaccination Act is an imperative necessity. Parliamentary legislation has declared its faith in vaccination, therefore the Act should embrace revaccination in addition. Only with such a system can there be freedom from small-pox epidemics. Voluntary, or panic vaccination is insufficient, as experience shows that a few cases of small-pox occurring in a large community are not sufficient to send the great bulk of the susceptible population to seek vaccination. There is much less opposition to vaccination in Scotland than in England. Yet although from April to the end of the year 1900 there had been over 400 cases of small-pox in Glasgow, the revaccinations in a population of more than three-quarters of a million amounted to only 8,001 persons over 10 years old. But in 1901 rapid increase of this disease caused rapid increase of vaccination, so that in that year the revaccinations ran up to over 400,000, not a single one of whom took small-pox. Among those who refused there were between April 1900 and May 1902 no fewer than 2,255 cases of small-pox. In Germany, since 1874, there has been compulsory revaccination of all school children in the twelfth year of life, as well as primary vaccination under two years. The result of this is, that small-pox epidemics are abolished from Germany, and only a few scattered deaths occur each year, mostly on the frontiers. A single revaccination cannot be regarded as absolute protection of the individual through the rest of his life. With regard to the Conscience Clause[5] in this country, no Act of Parliament has ever really been, or probably will ever really be, compulsory in the sense of forcing the vaccination of a child against the will of the parent. The present clause is faulty in two directions: (1) it allows a great many people to receive exemption certificates who are not true conscientious objectors ; (2) it allows refusals of certificates to other persons who are willing to prove their determined resistance to vaccination by every means which the law allows to them. It is also defective in leaving too much to the discretion of the magistrates. The latest returns show that in the provinces the conscientious objectors amount to about 5 per cent., and in London to about 1 per cent. With an amended Conscience Clause, and legislative obligation with regard to primary vaccination and to revaccination, it is likely that resort to exemption certificates would not be so great as if the present clause were retained. Parents would have less objection to vaccination at the age of 12 years, and as revaccination in time became a matter of routine among school children, the opposition would diminish. Resort to revaccination would become more general if the requirement became anything like a routine one in obtaining employment. There would be much less small-pox under a Revaccination Act with a Conscience Clause than under a primary Vaccination Act alone. Unless in presence of small-pox, the revaccination age should be made as late as is consistent with securing the vaccination of all children before leaving school. The Compulsory Education Acts would here come to the aid of an obligatory Revaccination Act. Experience in small-pox epidemics has shown that susceptibility to an attack has returned to a considerable percentage of persons between 10 and 15 years of age, and to an appreciable number even before 10 years of age. Should a certain age be fixed for revaccination, it ought always to be possible by order of the Local Government Board or otherwise, to temporarily lower the age limit in any given area until variola had disappeared from it. The mere possession of a vaccination certificate is no guarantee of the efficiency of the operation. In Gayton's 10,403 cases of small-pox, 2,085 had good vaccination marks, and the fatality rate was 3 per cent. ; 4,854 had indifferent marks, with a fatality rate of 9 per cent. ; 1,295 were alleged to be vaccinated but had no marks, with a fatality rate of 27 per cent., and 2,169 were unvaccinated, and had a fatality rate of 43 per cent. Government could insist that every vaccination certificate should in future state the number and area of marks, and also provide that a proper money payment is forthcoming for every certificate achieving a specified standard of success. A universal supply of lymph should be provided by the Government, for which there would be a regular demand if revaccination were made compulsory. Boards of Guardians[6] are not suitable vaccination authorities. For rural districts in England the County Council would be the best authority. Counties being large the councils ought to have such powers of devolution or allocation of work as would make for efficiency. County boroughs are already sanitary authorities and have sufficient areas for good administration. In London the same remark applies to the borough councils. Every sanitary authority should have full power to carry out vaccination and revaccination in times of emergency, as in presence of small-pox. The expenses in administering the Acts in county councils and large boroughs should be charged by rate over the whole area. Small-pox outbreaks under a vaccination and revaccination act with an exemption clause, would depend largely on the amount of exemption, and localities which had extensively resorted to the conscience clause should pay for their own small-pox, in the same way as would well vaccinated and revaccinated localities. Sir Michael Foster [7] whilst thinking that the conscientious objector will always remain, finds fault with the wording of the conscience clause. Government should more satisfactorily define the conscientious objector. The success of the struggle of vaccination against small-pox would depend upon the improved developments of the machinery of vaccination, and the early grappling with small-pox. A large proportion of the public objection to vaccination was due to the fact that the vaccination was badly done, with bad material, and badly cared for afterwards. The real success of vaccination depended largely on the utmost care being taken to secure the efficiency of the operation and the goodness of the material.

[4] Brit. Med. Jour., Jan 10, 1903. [5] Brit. Med. Jour., Jan. 17 1903. [6] Brit. Med. Jour., Jan. 24, 1903. [7] Public Health, April, 1903.

